# Two-Day Mohs Excision of Large Scalp Dermatofibrosarcoma Protuberans Reconstructed With a Rotation Flap

**DOI:** 10.7759/cureus.100911

**Published:** 2026-01-06

**Authors:** Jeison De Guzman, Zaineb Makhzoumi, Ronald P Silverman

**Affiliations:** 1 Department of Plastic and Reconstructive Surgery, University of Maryland School of Medicine, Baltimore, USA; 2 Department of Dermatology, University of Maryland School of Medicine, Baltimore, USA

**Keywords:** cutaneous sarcoma, dermatofibrosarcoma protuberans, mohs micrographic surgery, rotational flap, scalp reconstruction

## Abstract

Dermatofibrosarcoma protuberans (DFSP) is a rare, slow-growing, locally aggressive, cutaneous sarcoma with a high recurrence risk. Mohs micrographic surgery (MMS) offers superior margin control but presents significant reconstructive challenges in large scalp lesions. We present a 36-year-old male with a large (10 x 10 cm) DFSP of the frontal scalp requiring staged two-day MMS for complete clearance. Despite an extensive defect down to the periosteum and partial calvarium exposure, closure was achieved with a large rotational advancement flap with a split-thickness skin graft. This case highlights that even exceptionally large scalp DFSP lesions can be successfully excised under local anesthesia with MMS.

## Introduction

Dermatofibrosarcoma protuberans (DFSP) is a rare, slow-growing cutaneous soft-tissue sarcoma that originates in the dermis and progressively infiltrates the surrounding subcutaneous tissue. It most commonly arises on the trunk, followed by lower limbs, with only 10-17% of cases occurring in the head and neck region [1]. DFSP is usually asymptomatic, presenting as a painless, gradually enlarging, indurated, skin-colored, erythematous, or brownish-yellow, elevated plaque with irregular borders, often leading to delayed diagnosis. The tumor usually attaches to the overlying skin without involvement of deeper tissues such as fascia, striated muscle, periosteum, or bone. However, in cases of recurrence or long-standing lesions, invasion into these structures may occur, and up to 1% of cases metastasize [2]. Although metastasis is rare, the tumor is locally aggressive, with irregular finger-like projections that contribute to high recurrence rates if not completely excised [3].

Complete surgical excision with histologic margin control is the standard of care for DFSP, but complete removal can be difficult due to the tumor’s local spread and size. Mohs micrographic surgery (MMS) achieves the lowest recurrent rates while maximizing tissue preservation and is favored over wide local excision (WLE), particularly in the head and neck. Studies have shown MMS is advantageous over WLE in local recurrence rates and mortality, achieving tumor clearance with smaller margins (1 cm versus 3 cm) and better preservation of healthy tissue [4-6]. However, MMS becomes technically challenging with large lesions, particularly on the scalp, where the tumor can spread extensively with defects extending to bone. MMS is generally performed in the office setting under local anesthesia, which makes it challenging to fully resect large, complex tumors, which might be more commonly resected in the operating room under general anesthesia. 

We present a case of an extraordinarily large (10 x 10 cm) scalp DFSP, requiring a two-day staged MMS under local anesthesia to clear margins to the level of the calvarium, followed by subsequent successful reconstruction with a large rotational scalp flap and split-thickness skin graft performed in the operating room. This case demonstrates the feasibility of MMS for extensive DFSP on the scalp and the ability to close large scalp defects without microsurgical free flaps.

## Case presentation

A 36-year-old male with no prior history of facial trauma or radiation exposure presented with multiple enlarging nodules on the forehead. Over a decade earlier, the patient noted a small bump on the left frontal scalp, initially stable in size and tender only to deep palpation. Over time, the lesion increased in size and multiplied. Dermatology initially diagnosed benign cysts; however, the pathology was unclear.

**Figure 1:**
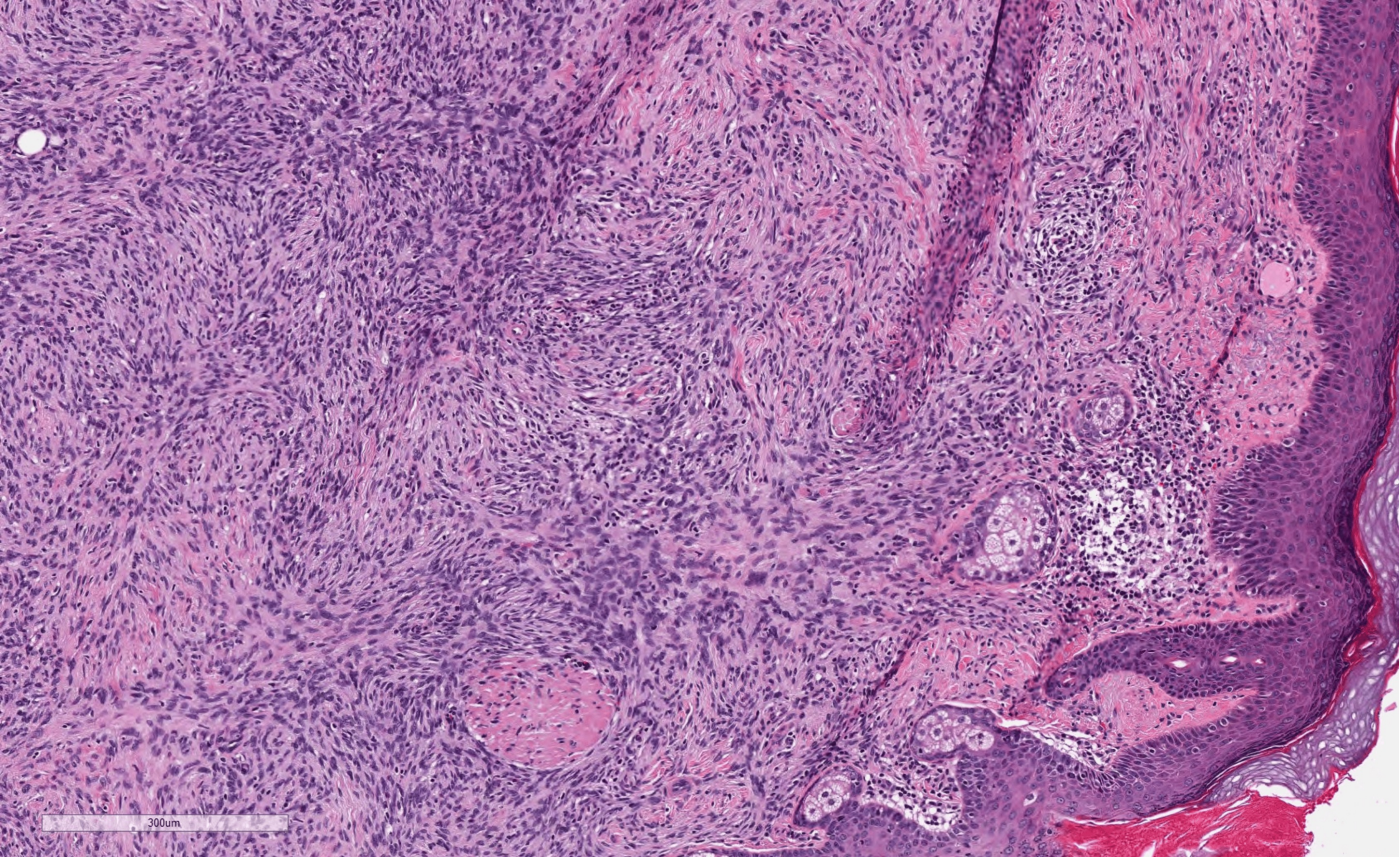
H&E revealing poorly demarcated neoplasms diffusely infiltrating the dermis and subcutis in a lace-like fashion.

**Figure 2:**
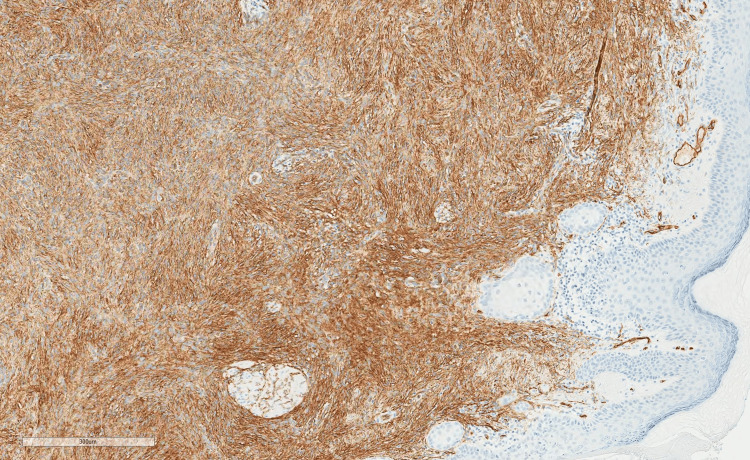
Infiltrative spindle cells positive for CD34.

**Figure 3:**
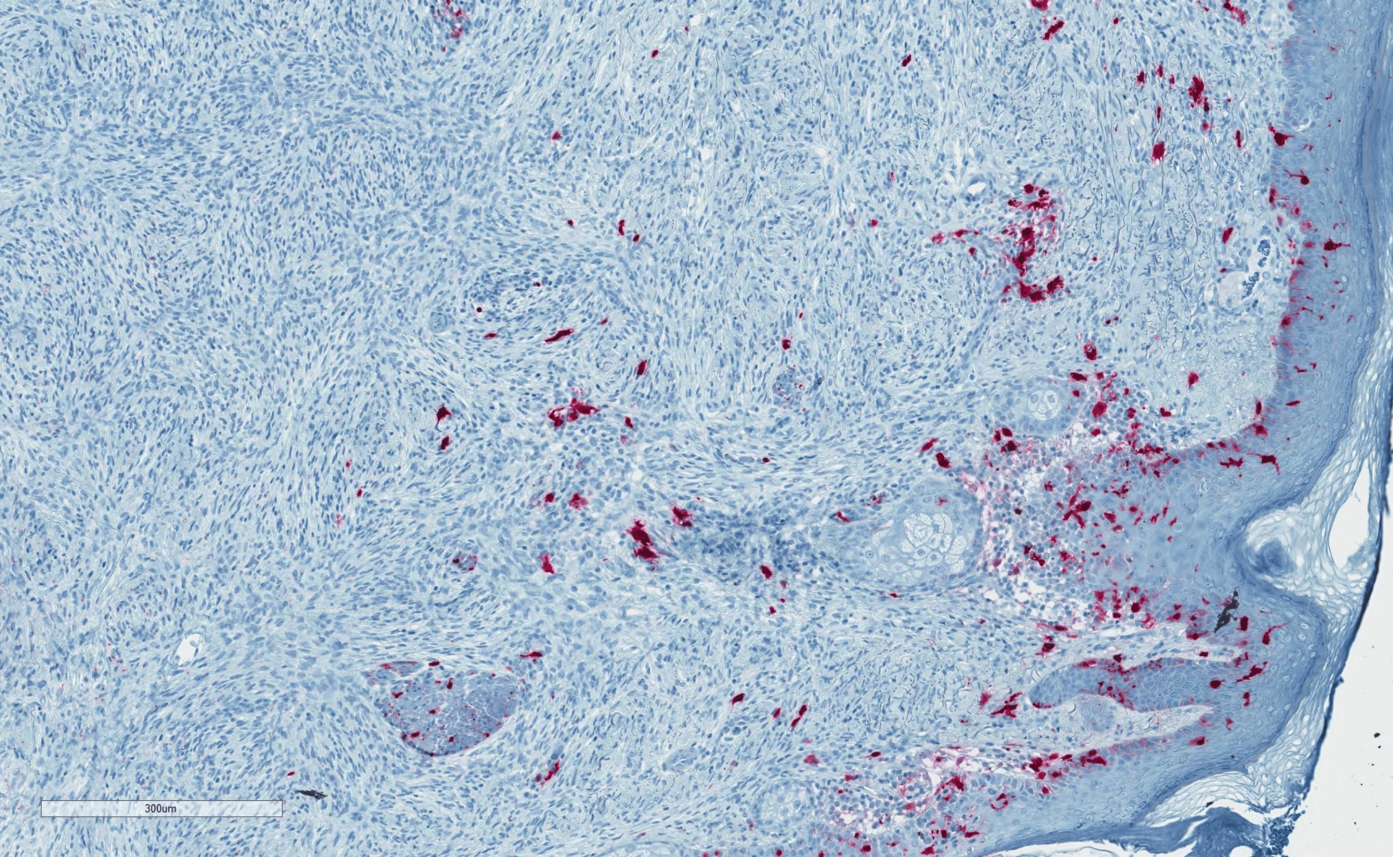
Infiltrative spindle cells negative for S100.

**Figure 4:**
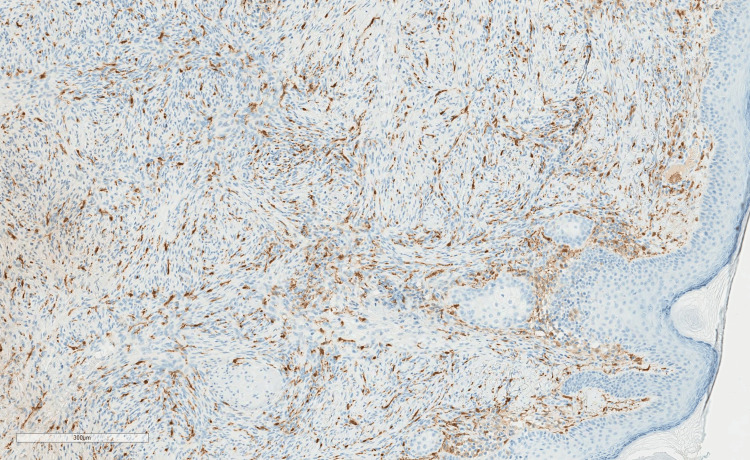
Infiltrative spindle cells negative for factor XIIIa.

The patient was referred to plastic surgery for surgical excision and biopsy. Adjacent to the hairline were two smooth, round, and mobile masses measuring one cm each, with several smaller, similar lesions along the scalp. Under local anesthesia, both lesions were excised. The final pathology revealed poorly demarcated neoplasms diffusely infiltrating the dermis and subcutis in a lace-like fashion. Infiltrative spindle cells were positive for CD34 and negative for S100 and Factor XIIIa, consistent with DFSP, with positive deep and lateral margins in both specimens (Figures 1-4).

Features of fibrosarcomatous transformation, including high-grade cytologic atypia, necrosis, or significantly increased mitotic activity, were not identified.

**Figure 5:**
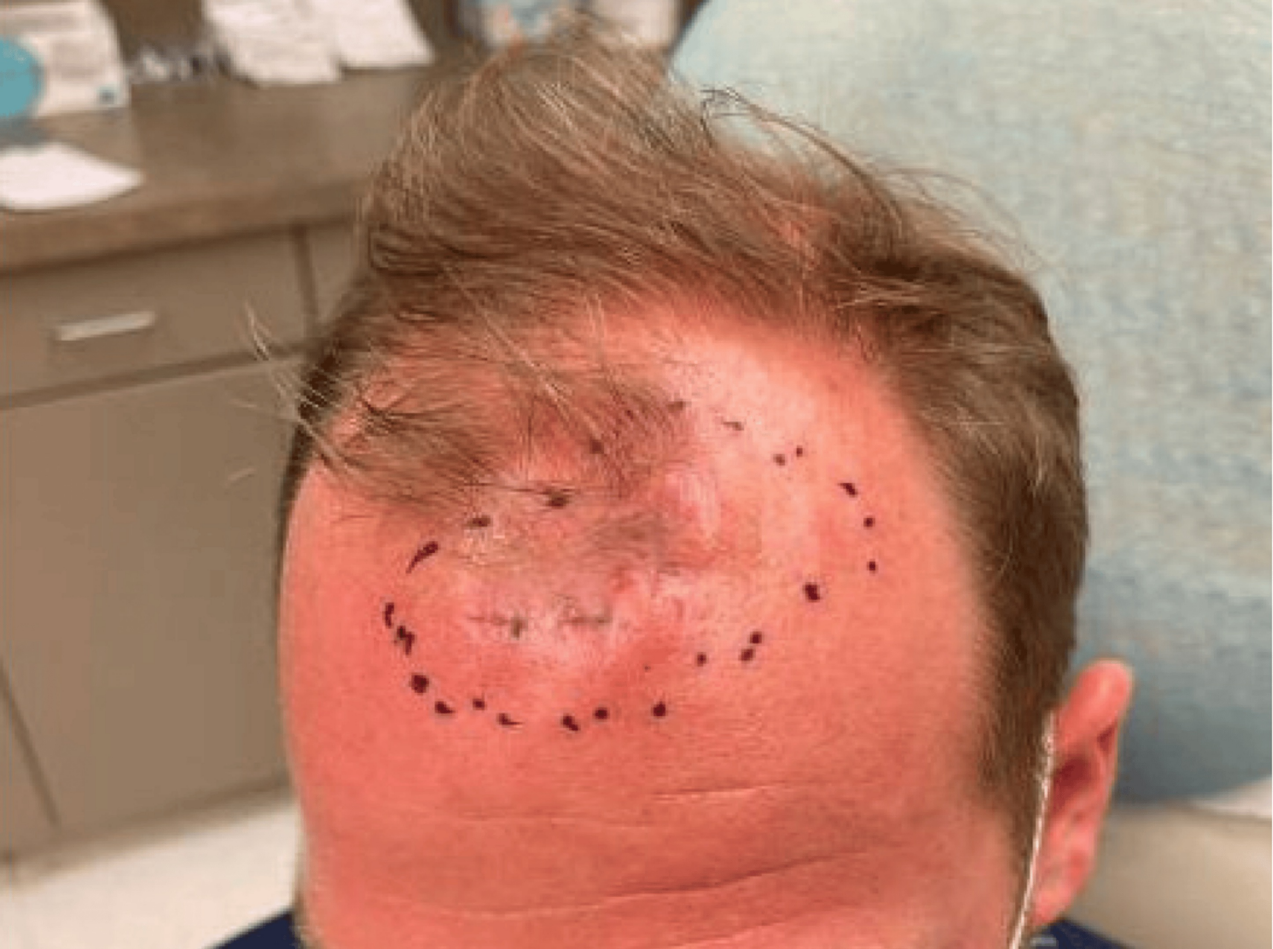
8 x 6 cm firm plaque on the scalp.

The patient was referred for MMS with dermatologic surgery. Two community-based Mohs surgeons declined to take the case due to concerns that the lesion would be too large to perform under local anesthesia in the office setting. A third Mohs surgeon at an academic center agreed to take on the case with the understanding that the defect would be reconstructed in the operating room by plastic surgery. Preoperatively, the lesion, which consisted of multiple adjacent nodules, measured 8 x 6 cm, poorly demarcated, and fixed to surrounding tissue (Figure 5).

**Figure 6:**
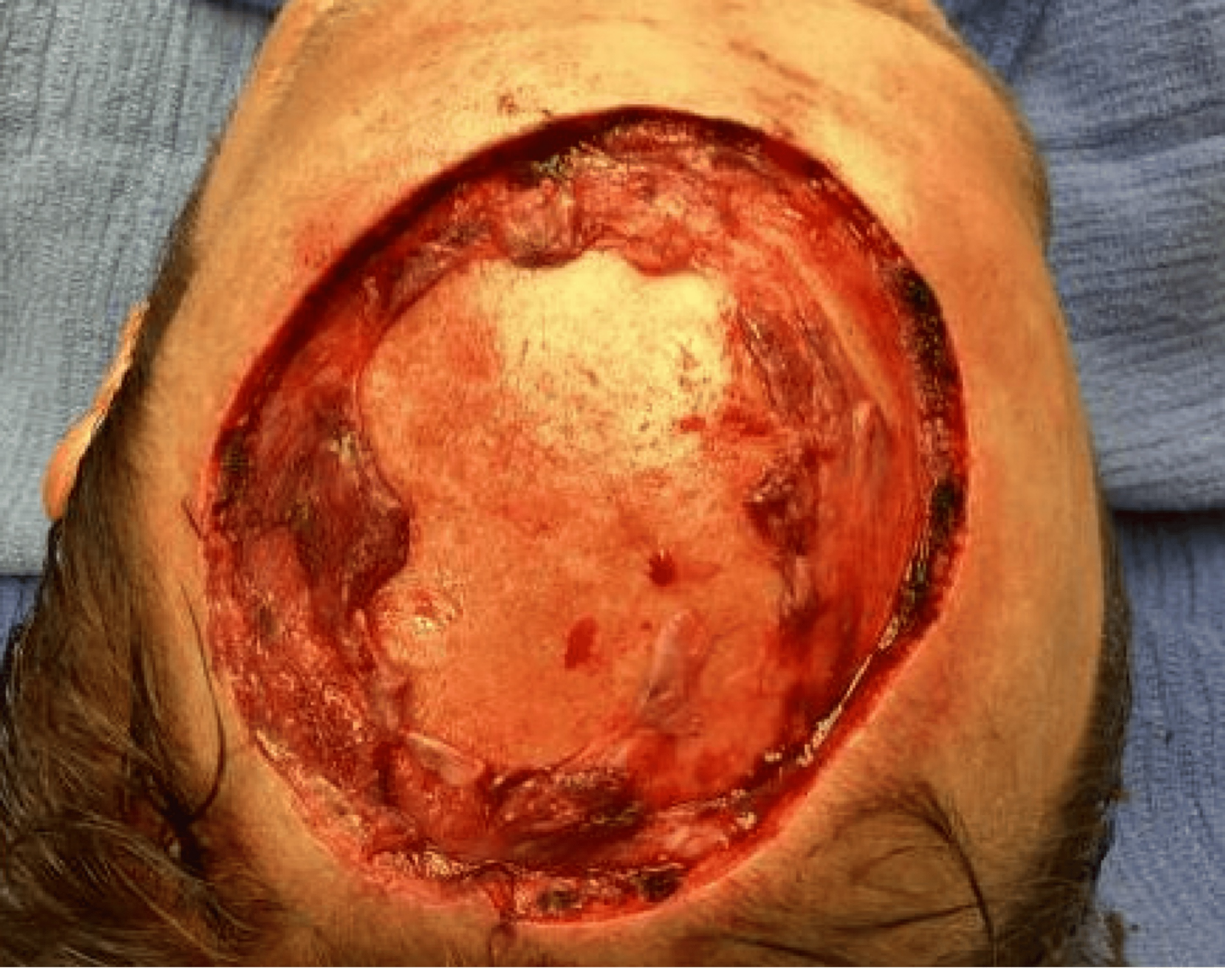
10 x 10 cm lesion following MMS excision of the scalp DFSP tumor. MMS: mohs micrographic surgery; DFSP: dermatofibrosarcoma protuberans

**Figure 7:**
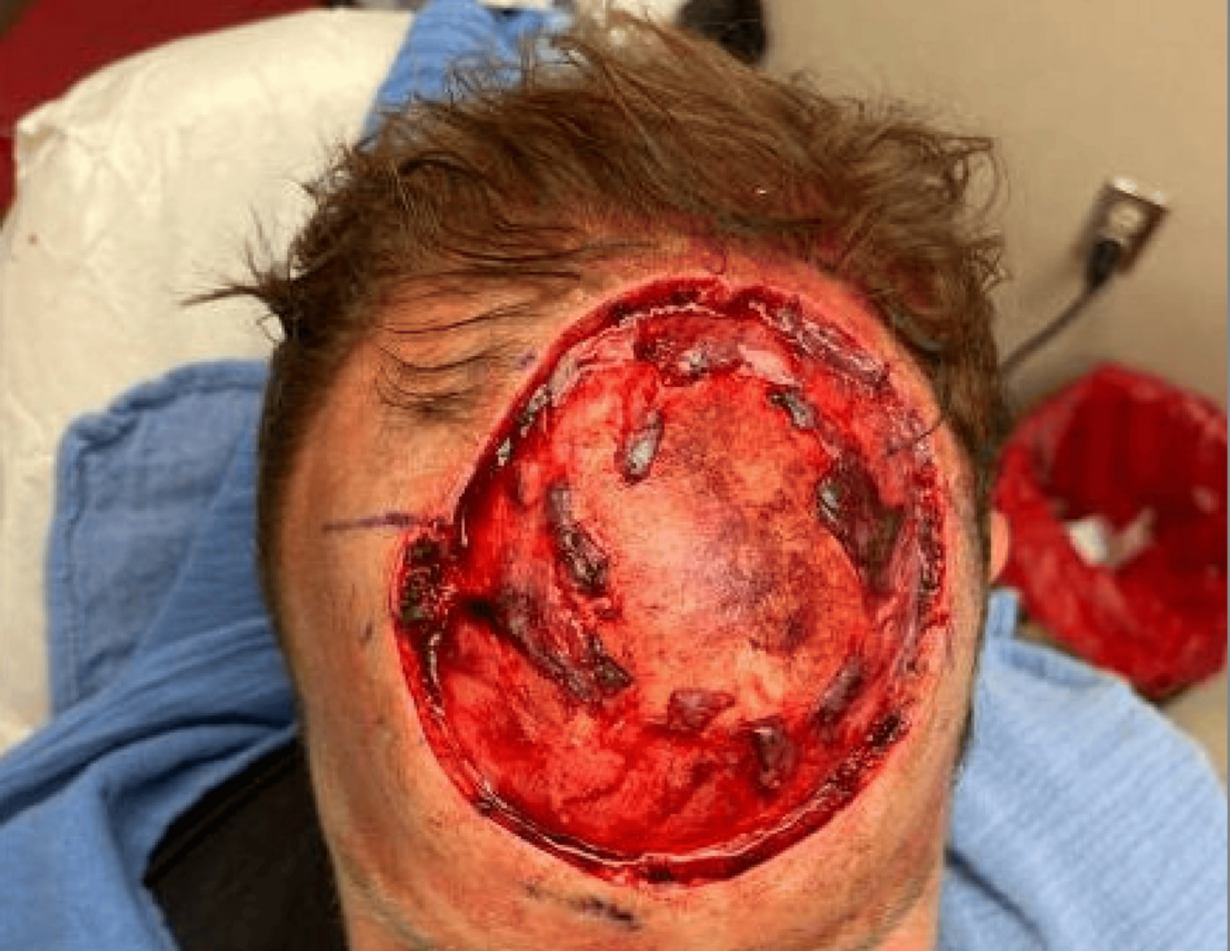
Three hours post-operative 10 x 10 cm scalp lesion.

During stage I MMS, an initial excision was made around the tumor with five mm margins. Following analysis of all frozen sections, the microscopic tumor persisted in 8/10 specimens. However, during cauterization after tumor extirpation, the patient experienced a vasovagal episode. Medical evaluation- including EKG and CT head without contrast- revealed normal sinus rhythm and no intracranial pathology. He returned the following day to complete MMS. Tissue from stage II showed a microscopic tumor in 4/8 frozen sections. The patient returned to the operative suite for a final Mohs stage, after which negative margins were achieved in 6/6 specimens. The final defect measured 10 x 10 cm, extending down to the calvarium (Figures 6-7).

**Figure 8:**
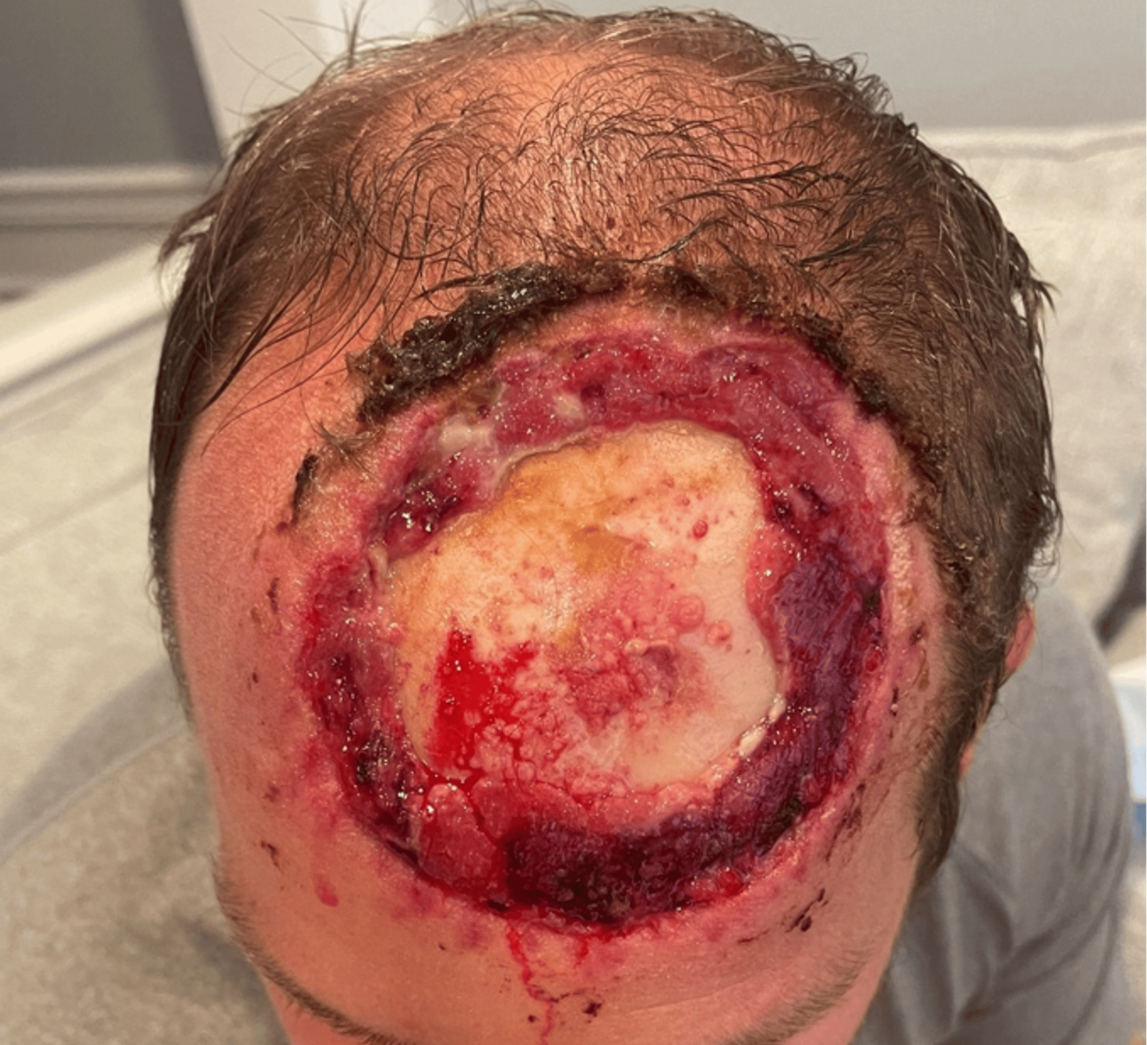
After removal of integra demonstrating partial take preoperatively before scalp rotation.

The next day, the patient underwent temporary closure with the plastic surgery service. Under general anesthesia, the defect was debrided and covered with an Integra® bilayer dermal regeneration template (Integra Lifesciences, Princeton, NJ), secured with a bolster dressing. After three weeks, the template had only partially taken (Figure 8).

**Figure 9:**
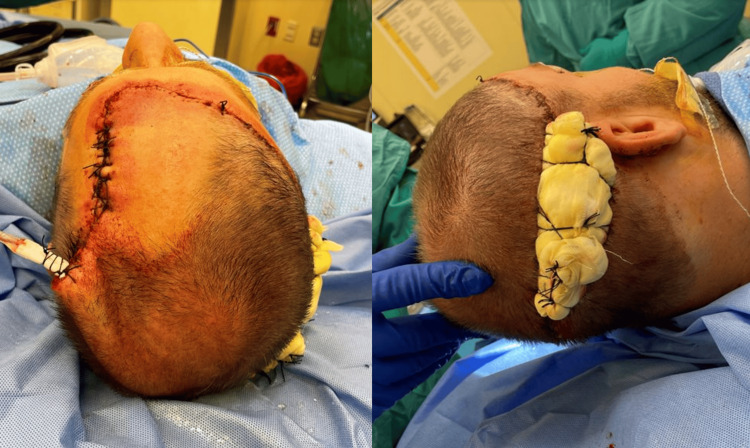
Intraoperative after performing scalp rotation; skin graft under xeroform bolster.

A rotational scalp flap measuring 300 cm^2^ was advanced to close the majority of the defect, with a 40 cm^2^ split-thickness skin graft (STSG) from the right thigh used to close the donor site on the posterior/lateral scalp (Figure 9).

**Figure 10:**
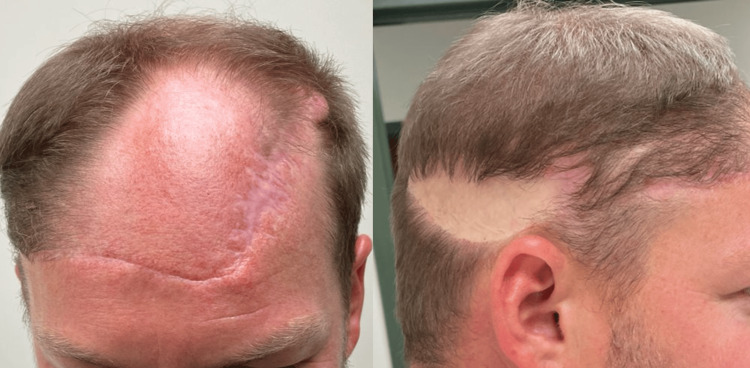
One-year post-operative from the scalp rotational flap.

The patient’s postoperative course was uncomplicated. On post-op day nine, the skin graft demonstrated near-complete take, and the flap remained viable without necrosis. One year after reconstruction, he elected for revision surgery (Figure 10).

**Figure 11:**
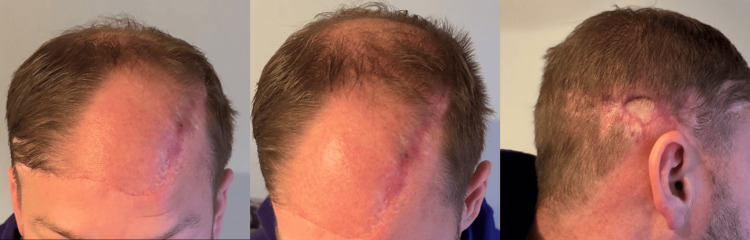
Six weeks post excision of the skin graft with a 2 cm x 1.5 cm residual skin graft.

The vast majority of the STSG of the scalp was excised and replaced with scalp flap advancement from the posterior scalp (Figure 11).

Pathology from both excised sites demonstrated scar and surgical site changes without recurrent tumor.

**Figure 12:**
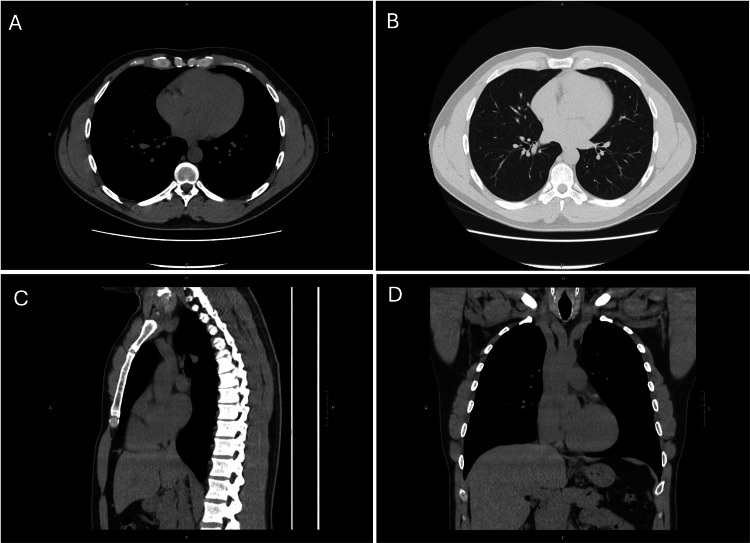
Two-year surveillance CT chest without contrast showing no evidence of intrathoracic metastasis. A: soft tissue body axial, B: lung axial, C: soft tissue body sagittal, D: soft tissue body coronal

Follow-up with dermatology showed a well-healed circumferential scalp scar with no evidence of recurrence. Surveillance CT chest two years postoperatively showed no evidence of metastatic disease (Figure 12).

The patient continues routine follow-up with dermatology and plastic surgery, with plans for continued long-term surveillance given the risk of late local recurrence in DFSP.

## Discussion

DFSP is a rare, slow-growing, locally aggressive sarcoma of the dermis with a tendency for subclinical spread. Head and neck involvement accounts for about 10% of cases, with even fewer presenting on the scalp [1]. Anatomical constraints in scalp DFSP include limited tissue mobility, proximity to cranial structures, and high cosmetic visibility, making complete excision particularly challenging.

Traditional WLE has been associated with local recurrence rates of up to 60%, largely due to DFSP’s finger-like projections extending beyond the clinically visible margin and irregular tumor shapes [7]. Studies have demonstrated the superiority of MMS over WLE in local tumor control [8-10]. MMS is advantageous in controlling the tumor burden microscopically while sparing uninvolved tissue- an especially important consideration in cosmetically and functionally sensitive areas such as the scalp. However, MMS is laborious, technically demanding, expensive, and time-consuming, particularly in multifocal, infiltrative DFSP of the scalp, and is generally performed under local anesthesia [11]. For this reason, many large scalp lesions like the case presented here are treated with a wide excision in the operating room, rather than MMS. In this case, MMS was instrumental in achieving complete tumor clearance. Initial wide local excision biopsies demonstrated positive deep and lateral margins, consistent with DFSP’s subclinical finger-like extensions. The first MMS stage revealed a persistent microscopic tumor in eight of 10 frozen sections, reflecting extensive subclinical spread. MMS cleared all margins after multiple stages. The final defect measured 10 x 10 cm, extending down to the calvarium, underscoring the aggressive local nature of DFSP and the challenge in scalp preservation.

Reconstruction after large scalp resections requires balancing durable coverage, contour restoration, and aesthetic integration. Options include primary closure, skin grafting, local rotational or advancement flaps, and free tissue transfer, with selection driven by defect size, location, and patient-specific factors. A recent systematic review of scalp DFSP patients showed the median defect size was 55.8 cm^2^ (interquartile range 48-112 cm^2^). Most required local (41.8%) or free flap (27.8%) reconstruction [10]. Local flaps are considered the workhorse for moderate defects (<45 cm^2^), providing hair-bearing, durable, and vascularized soft-tissue coverage. Free flap reconstruction is typically considered for defects larger than 45 cm^2^; the gracilis muscle or anterolateral thigh (ALT) flap for scalp defects <300 cm^2^, and the latissimus dorsi myocutaneous (LDM) flap for larger defects [12].

In our patient, a staged approach was utilized. Initial placement of a dermal regeneration template with the goal of providing some vascularized coverage over the calvarium, and therefore leaving the option for skin grafting if required. Three weeks following the dermal regeneration template, it was clear that the template had only partially taken, and there would be a need for additional reconstruction.

After extensive discussions with the patient about options, risks, and benefits, it was decided to proceed with a large rotational scalp flap with a skin graft to the donor site. In the operating room under general anesthesia, a large rotational scalp flap was mobilized to cover the tumor defect, utilizing adjacent scalp tissue to restore contour and preserve hair-bearing skin. The flap donor site was closed with an STSG harvested from the thigh, balancing donor site morbidity and providing adequate coverage.

This combination of local flap and skin grafting allowed for tension-free closure of a complex 300 cm^2^ defect without resorting to a free flap. Later revision surgery addressed residual contour irregularities and aesthetic concerns by excising most of the non-hair-bearing skin graft and replacing it with adjacent hair-bearing scalp tissue, improving cosmesis while maintaining stable coverage.

## Conclusions

This case highlights the clinical challenge and management complexity of scalp DFSP. MMS proved essential in achieving complete tumor clearance despite extensive microscopic infiltration beyond clinical margins. The combination of staged Mohs micrographic resection, dermal regeneration template placement, and rotational scalp flap with skin grafting facilitated a durable, functional, and aesthetically acceptable reconstruction of a large scalp defect while preserving hair-bearing tissue. Long-term surveillance remains critical due to the risk of local recurrence.
